# Late Recurrence of Renal Cell Carcinoma Presenting With Skeletal Muscle Metastases: A Case Report

**DOI:** 10.7759/cureus.96223

**Published:** 2025-11-06

**Authors:** Sara Y Amro, Mutaz Kassas, Madiha Hijazi, Mustafa Natout

**Affiliations:** 1 Radiology, American University of Beirut Medical Center, Beirut, LBN; 2 Nuclear Medicine, Institut Jules Bordet, Hôpital Universitaire de Bruxelles, Université Libre de Bruxelles, Brussels, BEL

**Keywords:** case report, hypervascular, late-onset, renal cell carcinoma, skeletal metastasis

## Abstract

Metastatic spread from renal cell carcinoma (RCC) can occur in unusual locations, many years after curative nephrectomy. We report the case of a 71-year-old male patient who developed multiple asymptomatic skeletal muscle metastases from RCC, seven years after curative nephrectomy. The lesions were discovered incidentally on CT angiography performed for peripheral artery disease and demonstrated hypervascularity with prominent feeding arteries, consistent with metastatic RCC. Additional pancreatic and pulmonary metastases were also identified on CT. The patient declined further intervention due to poor health status. This case highlights the potential for late, asymptomatic, and unusual metastatic sites in RCC, emphasizing the importance of considering extended surveillance in long-term survivors.

## Introduction

Renal cell carcinoma (RCC) constitutes 80% of all primary renal malignancies and accounts for 4% of all adult malignancies [1]. The lungs, bones, lymph nodes, and liver are the most common metastatic sites for RCC [2]. Less frequent or atypical sites include the pancreas, thyroid, skin, and skeletal muscle, the latter being exceedingly rare, occurring in less than 1% of patients [3,4]. 

In patients who initially present without disseminated disease at the time of nephrectomy, most recurrences develop within the first two years after surgery; however, a subset experiences late relapse beyond five years, a well-recognized biological behavior of RCC. A five-year disease-free interval is therefore commonly used to define late recurrence and serves as a practical and clinically relevant threshold for distinguishing this population [5]. 

The classic triad of flank pain, gross hematuria, and a palpable mass, once characteristic of RCC, is now less frequent, as most cases are incidentally detected through imaging [6]. In metastatic RCC, clinical manifestations vary according to the sites of metastatic involvement. In particular, skeletal muscle metastases from RCC often present as a painless or slowly enlarging soft-tissue mass, though some patients may experience localized pain, swelling, or tenderness depending on the depth and site of involvement [7]. The differential diagnosis for skeletal muscle metastasis includes benign soft-tissue lesions such as intramuscular lipoma or hematoma, primary soft-tissue sarcoma, abscess or inflammatory myositis, and metastases from other primary malignancies such as lung, colon, or melanoma. Late recurrence of RCC is diagnosed through a combination of clinical suspicion, imaging, and histopathologic confirmation. Cross-sectional imaging, typically contrast-enhanced CT or MRI, is used to detect new lesions during follow-up or incidentally, while biopsy with immunohistochemical staining confirms renal origin and distinguishes recurrence from other pathologies [6]. 

As surgical excision of isolated metastases can improve disease-free survival (five-year survival of 32.5% with complete metastasectomy vs. 12.4% without [8]), it remains essential to maintain a high index of suspicion for skeletal muscle metastases in RCC, as early recognition and intervention can significantly influence patient outcomes. In recurrent RCC, survival outcomes vary markedly with the timing of recurrence. Patients with late recurrence demonstrate significantly better prognosis, with a five-year cancer-specific survival rate of approximately 73.7%, compared to 41.1% for early recurrence, and 27.0% for synchronous metastatic disease, reflecting the more favorable clinicopathological characteristics of late-recurrent tumors [9]. 

The treatment of metastatic RCC has shifted toward systemic immunotherapy-based combinations, with programmed death-1 (PD-1) inhibitors paired with Vascular Endothelial Growth Factor Receptor (VEGFR)-targeted agents (such as pembrolizumab-lenvatinib, pembrolizumab-axitinib, or cabozantinib-nivolumab), now representing standard first-line options across risk groups. Cytoreductive nephrectomy and metastasectomy may still be considered in carefully selected patients with low-volume or oligometastatic disease following multidisciplinary review, while surgery is generally avoided in poor-risk or early-relapsing cases where systemic therapy offers superior outcomes [6].

Given this potential for delayed and unusual spread, clinicians should remain vigilant for rare metastatic sites such as skeletal muscle. We present an asymptomatic, multifocal skeletal muscle metastasis from RCC discovered incidentally on imaging seven years after curative nephrectomy, highlighting the importance of considering such atypical presentations in long-term RCC survivors.

## Case presentation

A 71-year-old man with a history of right RCC status post curative nephrectomy seven years prior presented to the hospital with a non-healing ulcer at the site of a recent left foot amputation. He was known to have long-standing type 2 diabetes mellitus complicated by peripheral arterial disease, which had led to amputations of the left fourth and fifth toes two months earlier. His medical history was also significant for an ischemic stroke one year ago, resulting in aphasia, which limited his ability to communicate symptoms. He had no known family history of malignancy.

In the month before presentation, the patient’s mobility had been severely limited, and he was largely bedridden at home. According to his family, he occasionally attempted to get out of bed unassisted, resulting in several falls. Approximately one week prior to admission, he sustained a fall that was followed by persistent pain in the right hip. At presentation, his chief complaints were a non-healing left foot wound (at the amputation site) and right hip pain. He was admitted for management of the foot ulcer and was scheduled for a left lower-extremity femoropopliteal bypass to improve blood flow for wound healing. On examination, point tenderness was noted over the right proximal femur, with the right leg appearing slightly shorter than the left and externally rotated. Given these findings and the history of recent trauma, a plain radiograph of the pelvis and right hip was obtained, which revealed a non-displaced right intertrochanteric femur fracture. Orthopedic surgery was consulted, and the patient was scheduled for right hip fracture fixation. As such, the vascular bypass surgery was postponed, with the orthopedic repair prioritized first. At the time of his presentation, the patient was considered to be in oncologic remission and was not receiving any adjuvant chemotherapy or targeted therapy.

On routine lab studies, creatinine, estimated glomerular filtration rate (eGFR), and blood urea nitrogen (BUN) were all within normal range (creatinine:0.9 mg/dL (reference range: 0.6-1.2 mg/dL); eGFR: 86 mL/min/1.73m^2 (^reference range: >60mL/min/1.73m^2^); BUN: 11 mg/dL (reference range: 8-25 mg/dL)), but urine analysis demonstrated trace hemoglobin and few red blood cells. No liver enzymes or creatine phosphokinase (CPK) levels were obtained. The patient’s laboratory results are summarized in Table 1, including complete blood count, basic metabolic panel, and urinalysis.

**Table 1:** Patient's key laboratory findings CBC, complete blood count; WBC, white blood cell; RBC, red blood cell; HGB, hemoglobin; HCT, hematocrit; BMP, basic metabolic panel; BUN, blood urea nitrogen;  eGFR, estimated glomerular filtration rate; CRP, C-reactive protein; hpf, high power field.

Test on admission	Result	Reference range
CBC		
WBC Count	10,700 /µL	4,000–11,000 /µL
RBC Count	4.67 ×10⁶/µL	4.5–5.9 ×10⁶/µL
HGB	13.4 g/dL	13.5–17.5 g/dL
HCT	41%	41–53%
Platelets	329,000/µL	150,000–450,000/µL
Neutrophils	78%	40–75%
Lymphocytes	12%	20–45%
BMP		
Creatinine	0.9 mg/dL	0.6–1.2 mg/dL
eGFR	86 mL/min/1.73m²	>60 mL/min/1.73m²
BUN	11 mg/dL	8-25 mg/dL
Sodium	134 mmol/L	135–145 mmol/L
Potassium	4.6 mmol/L	3.5–5.1 mmol/L
Calcium	9.0 mg/dL	8.5–10.5 mg/dL
Inflammatory Markers		
CRP	95.7 mg/L	<5 mg/L
Urinalysis		
Color	Yellow	—
Clarity	Light turbid	Clear
Specific gravity	1.014	1.005–1.030
pH	7	4.5–8.0
Protein	Trace	Negative
Glucose	3+	Negative
Ketones	1+	Negative
Hemoglobin	Trace	Negative
RBC	2–4 /hpf	0–2 /hpf
WBC	Rare	0–5 /hpf
Crystals	Many (amorphous)	Absent

Additionally, the patient had marked weight loss of approximately 15 kgs in three years. On physical exam, no flank tenderness, abdominal mass, organomegaly, supraclavicular lymphadenopathy, or pallor were noted. The patient was mildly hypertensive on admission (157/57 mmHg, reference range: 90-120/60-80 mmHg), but all remaining vital signs were within normal range.

**Figure 1:**
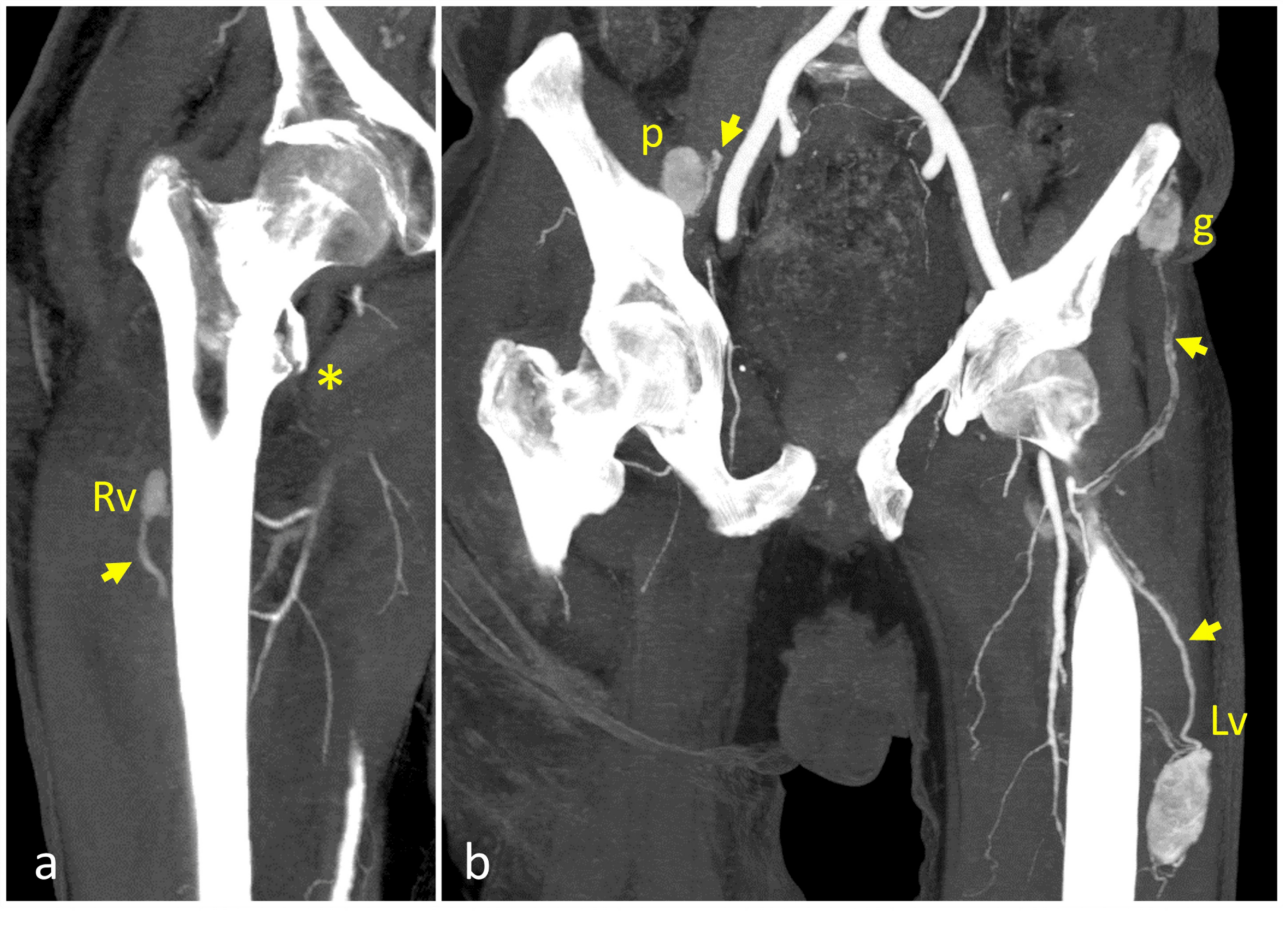
Arterial-phase CT with maximum intensity projection (MIP) of the pelvis and hips Image shows multiple hypervascular intramuscular masses with large feeding arteries and a right intertrochanteric fracture (*). Muscles highlighted include right vastus intermedius (Rv), right iliopsoas (p), left gluteus medius (g), and left vastus lateralis (Lv) with their corresponding feeding arteries (short arrows).

As part of the pre-operative assessment for the planned bypass, a contrast-enhanced computed tomography angiography (CTA) of the lower extremities was performed. This study revealed several intramuscular, arterially hyperenhancing soft-tissue lesions with prominent feeding arteries, findings consistent with hypervascular metastases (Figure 1).

Five discrete intramuscular lesions were identified: a 5.1 × 2.4 × 2.2 cm lesion in the left vastus lateralis muscle, a 2.7 × 2.1 × 1.7 cm lesion in the left gluteus medius, a 2.6 × 1.8 × 1.5 cm lesion in the right iliacus, a 1.0 × 0.8 cm lesion in the left iliacus, and a 1.1 × 0.8 cm lesion in the right vastus intermedius. The previously noted femoral fracture was visualized on the same scan, showing minimal separation of the major fractured fragments along with minimal displacement.

**Figure 2:**
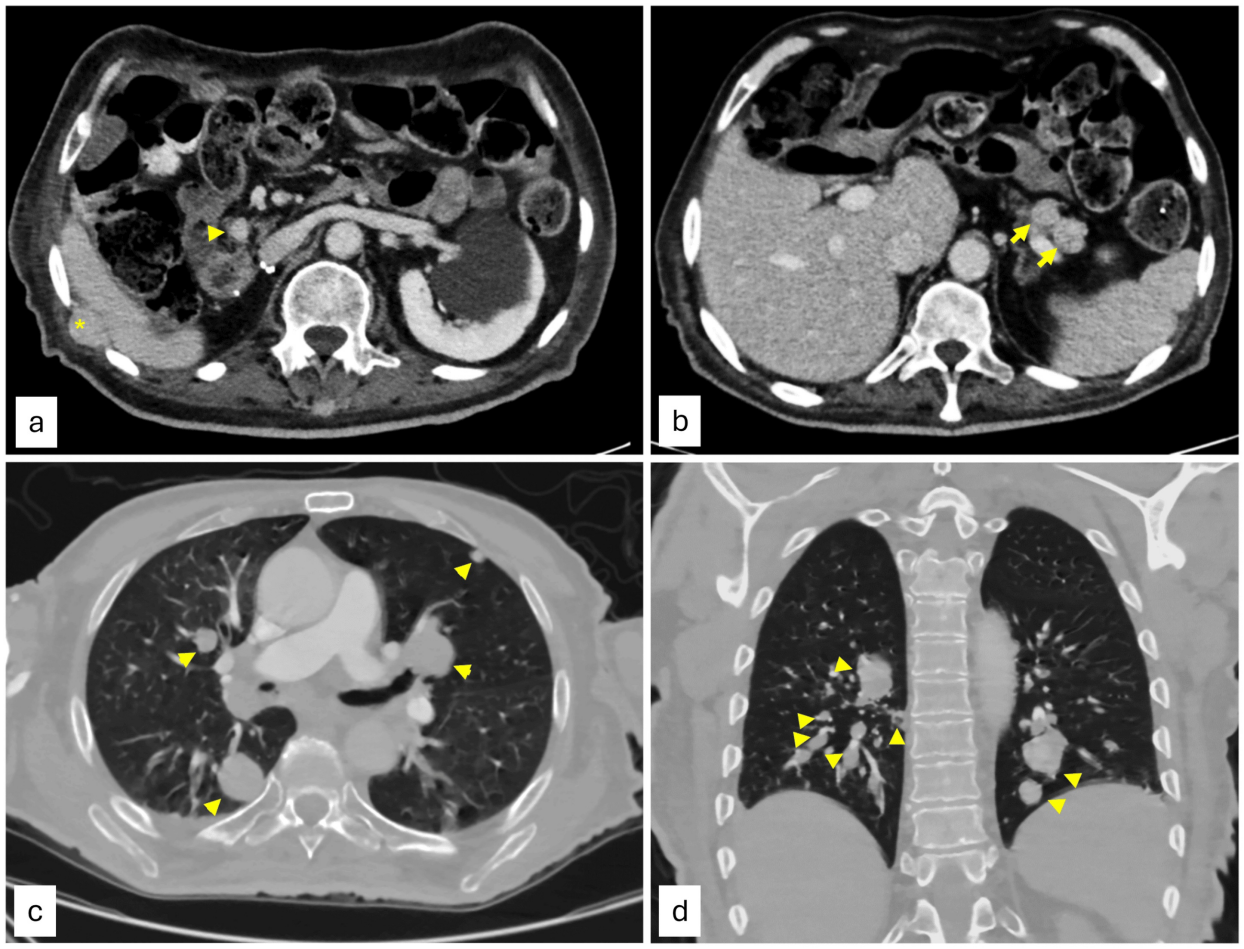
Contrast-enhanced CT scan images (a) & (b) Axial contrast enhanced CT scan images of the upper abdomen showing hyper-enhancing lesions involving the pancreatic head (arrowhead), right 10th intercostal space (*) and pancreatic tail (short arrow); CT scan images of the lungs in the (c) axial & (d) coronal planes, demonstrating multiple bilateral pulmonary nodules (short arrows).

During his hospital stay, the patient had an episode of desaturation, and subsequently underwent an urgent CT angiography of the chest to rule out pulmonary embolism, which additionally revealed innumerable bilateral metastatic pulmonary nodules of variable sizes (range 1~2.5 cm), suggestive of metastases (Figures 2c, 2d).

Subsequently, a follow-up contrast-enhanced CT scan of the abdomen and pelvis was obtained for RCC staging after evaluation by the oncology team, and revealed no soft tissue lesions at the surgical bed excluding local disease recurrence. However, there were multiple enhancing lesions along the pancreas suggestive of metastatic deposits. Additional lesions in the abdominal wall were present, including the right 10th intercostal space (Figures 2a, 2b).

Although a CT-guided lung biopsy was scheduled to confirm the metastases, the patient and his family refused to proceed with it and preferred to pursue oncological treatment in his home country, considering the patient’s poor overall status and newly discovered metastatic disease. A chronological summary of the patient’s clinical course, from nephrectomy to the current hospitalization, is provided in Table 2. 

**Table 2:** Timeline of key events in the patient’s course RCC, renal cell carcinoma, CTA, Computed Tomography Angiography.

Time	Clinical event
7 years prior to presentation	Right radical nephrectomy for Stage I RCC (no recurrence on follow-up).
Day of admission	Presented with pre-necrotic, non-healing ulcer at left foot amputation site (diabetic foot ulcer). Noted to have persistent right hip pain after recent fall.
Hospital day 1	Physical exam indicated right hip injury; pelvic X-ray confirmed a right intertrochanteric femur fracture. Orthopedic surgery (hip nailing) planned; vascular bypass surgery postponed.
Hospital day 2	Pre-operative CTA of lower extremities (for bypass planning) revealed multiple hypervascular intramuscular lesions in pelvic and thigh muscles (suspicious for metastases).
Hospital day 3	Episode of oxygen desaturation; CT pulmonary angiography ruled out embolism but revealed numerous bilateral lung nodules (1–2.5 cm) consistent with pulmonary metastases.
Hospital day 4	Contrast CT of abdomen and pelvis done for staging showed multiple pancreatic lesions and a right lateral abdominal wall lesion, consistent with metastatic RCC.
Hospital day 5	Multidisciplinary discussion and oncology evaluation. CT-guided biopsy was offered for diagnostic confirmation, but the patient declined further invasive procedures.
Discharge plan	Given extensive metastatic disease and poor functional status, the patient opted for palliative management and follow-up with oncology in his home country.

## Discussion

Metastases to skeletal muscles are an uncommon finding in patients with RCC, with a reported incidence in the literature of less than 1% [3]. Various postulations have been suggested to explain why skeletal muscles are an unfavorable site for metastasis, including the presence of muscle-derived protease inhibitors in the extracellular muscle matrix, lactic acid production in muscle cells that results in angiogenesis resistance, local temperature fluctuations, and muscular contractions that dislodge tumor cells [10].

Metastases from RCC are notoriously unpredictable and widespread, even after curative nephrectomy. They may occur at distant sites several years post-surgery without suggestion of local disease recurrence [11], as evident in our patient. The most reported sites of RCC metastases include the lungs, bone, lymph nodes, and liver, in this order [2]. Patients with skeletal muscle metastasis usually present asymptomatically until the mass enlarges to cause pain, swelling, or obvious protrusion. Routine oncological imaging for RCC typically excludes extremities, which are only imaged if associated symptoms are present. This could additionally contribute to the low incidence of RCC metastases to skeletal muscle reported in the literature. The mainstream adoption of positron emission tomography/computed tomography (PET/CT) as a modality for follow-up on malignancies is likely to increase the detection rate of such metastases [12].

Radiologically, skeletal muscle metastases from RCC are characteristically hypervascular and supplied by large feeding arteries, features that were evident in the intramuscular lesions in our patient. RCC metastases to skeletal muscle usually appear on CT as intramuscular soft-tissue masses that may be oval, round, irregular or lobular [13]. They are characteristically show avid contrast enhancement, which is frequently heterogeneous or manifests as a peripheral rim if central necrosis is present. Prominent feeding arteries or serpentine vessels may be seen supplying the tumor on arterial-phase imaging, reflecting its rich vascular supply [14]. On unenhanced CT, these metastases can be nearly isoattenuating to normal muscle, so intravenous contrast is essential for their detection [13]. In a patient with prior RCC, the finding of a new hyperenhancing intramuscular mass is highly suggestive of metastatic disease.

Several cases of RCC metastases to skeletal muscles have been previously reported in the literature [6]. No consensus is established on whether the muscles of the limbs or of the trunk are the most common site of skeletal muscle metastases [15]. The lack of a preferred metastasis site is likely due to the hematogenous spread of RCC rather than by direct invasion, a finding corroborated in our case by the presence of large feeding arteries supplying the hypervascular lesions [16].

While biopsy is the gold standard for confirming metastatic disease, in this case, it was not pursued due to the patient’s condition and preference. Nonetheless, the convergence of several factors including a prior history of RCC, the presence of multiple hypervascular intramuscular lesions with feeding arteries, and synchronous pancreatic and pulmonary metastases all makes alternative diagnoses highly unlikely. These radiologic and clinical findings, taken together, strongly support the interpretation that the lesions represent metastatic RCC despite the absence of tissue confirmation.

As noted previously, the typical presentation in the literature is that of a solitary, palpable, solid mass often associated with pain and discomfort [17]. Damron et al. reported that RCC metastases in soft tissues, including skeletal muscle, almost always present as solitary masses after a variable interval from the initial diagnosis of RCC [18]. However, this observation may be influenced by reporting bias, as biopsy is more often performed for isolated lesions, whereas multiple concomitant metastases often do not individually warrant biopsy and thus may be underrepresented. In contrast, the detection of distant skeletal muscle metastases in our patient was entirely incidental, without visible or palpable swelling, and he presented with multiple intramuscular lesions along with abdominal and pulmonary metastases. This pattern differs from the solitary descriptions emphasized in the literature, and highlights the unpredictable behavior of RCC metastases in both presentation and location and the need for life-long follow-up.

## Conclusions

This case illustrates that RCC can recur as late as seven years after nephrectomy, presenting with multifocal, hypervascular skeletal muscle lesions that were asymptomatic and discovered only incidentally on imaging. Our patient demonstrated radiological findings consistent with RCC metastases, namely arterial hyperenhancement and large feeding arteries supplying the hypervascular lesions. The concomitant finding of pancreatic and pulmonary metastases in our patient further emphasizes the unpredictable and widespread nature of late RCC recurrence. Although histopathological confirmation was not obtained due to the patient’s refusal of biopsy, the characteristic radiologic features and clinical history strongly supported the diagnosis of metastatic RCC. Overall, these observations highlight the limitations of relying on symptoms to detect recurrence and reinforce the need to consider prolonged, individualized surveillance strategies for RCC survivors.
